# Comprehensive Analysis and Identification of the Human STIM1 Domains for Structural and Functional Studies

**DOI:** 10.1371/journal.pone.0053979

**Published:** 2013-01-08

**Authors:** Jonathan How, Ai Zhang, Margaret Phillips, Aline Reynaud, Si Yan Lu, Lucy Xin Pan, Hai Ting Ho, Yin Hoe Yau, Albert Guskov, Konstantin Pervushin, Susana Geifman Shochat, Said Eshaghi

**Affiliations:** Division of Structural Biology and Biochemistry, School of Biological Sciences, Nanyang Technological University, Singapore, Republic of Singapore; University of Oldenburg, Germany

## Abstract

STIM1 is a Ca^2+^ sensor within the ER membrane known to activate the plasma membrane store-operated Ca^2+^ channel upon depletion of its target ion in the ER lumen. This activation is a crucial step to initiate the Ca^2+^ signaling cascades within various cell types. Human STIM1 is a 77.4 kDa protein consisting of various domains that are involved in Ca^2+^ sensing, oligomerization, and channel activation and deactivation. In this study, we identify the domains and boundaries in which functional and stable recombinant human STIM1 can be produced in large quantities. To achieve this goal, we cloned nearly 200 constructs that vary in their initial and terminal residues, length and presence of the transmembrane domain, and we conducted expression and purification analyses using these constructs. The results revealed that nearly half of the constructs could be expressed and purified with high quality, out of which 25% contained the integral membrane domain. Further analyses using surface plasmon resonance, nuclear magnetic resonance and a thermostability assay verified the functionality and integrity of these constructs. Thus, we have been able to identify the most stable and well-behaved domains of the hSTIM1 protein, which can be used for future *in vitro* biochemical and biophysical studies.

## Introduction

A primary route for calcium ion (Ca^2+^) entry across the plasma membrane is through store-operated channels that are widespread yet primordial among Ca^2+^ permeable ion channels [1]. The prototypic store-operated channel, as characterized in T-lymphocytes and mast cells, is the Ca^2+^ release-activated Ca^2+^ (CRAC) channel [2]. This channel is activated by the depletion of intracellular Ca^2+^ stores within the endoplasmic reticulum (ER) lumen caused by the release of the second messenger inositol-1,4,5-trisphosphate (IP_3_). STIM1 has been identified as the Ca^2+^ sensor and regulator of Ca^2+^ influx and CRAC channel function [2]–[4] and is an ER membrane protein with a single transmembrane helix. STIM1 consists of an N-terminal region, which contains two EF-hand Ca^2+^-binding motifs, that is located in the ER lumen; a sterile alpha motif (SAM) known to be involved in protein-protein interactions; one transmembrane domain; and a cytosolic C-terminus containing three coiled-coil regions, a Ser/Pro region and a Lys-rich domain. STIM1 senses the Ca^2+^ concentration inside the ER lumen through its EF-hand motif. Upon depletion of Ca^2+^ from the ER lumen, STIM1 loses its bound Ca^2+^, oligomerizes and interacts with the plasma membrane Ca^2+^ channel, Orai1, thereby activating the channel [1]. The activation of Orai1 is believed to occur through the interactions of residues 339–448, also known as the channel activation domain (CAD), within the coiled-coil region of the human STIM1 (hSTIM1) with the C-terminus of Orai1 [5]–[7]. Recently, the crystal structure of the CAD domain was reported, which was the first evidence that confirmed its coiled-coil nature [8]. The structures of the CAD and the N-terminal region of hSTIM1 [9] are the only high-resolution structures of this protein available to date. In fact, most of the current knowledge concerning the function of hSTIM1 is based on *in vivo* studies. This limited knowledge can be attributed to the nature of hSTIM1; it is a transmembrane protein with a large coiled-coil region (approximately 150 amino acids) and has evolved to oligomerize in order to reach its active state. In an attempt to produce large quantities of different domains of hSTIM1 using both an *Escherichia coli* (*E. coli*) and a *Saccharomyces cerevisiae* (S. *cerevisiae*) expression system, we noticed that many of the constructs were very unstable and either heavily aggregated or degraded. This result would further explain the lack of information available regarding large-scale preparation of hSTIM1 that hampers the detailed biochemical, biophysical and characterization studies of hSTIM1 using *in vitro* systems.

Challenges in the production of recombinant proteins may be correlated to the amino acid composition of the primary sequence. Designing multiple constructs of a single protein often leads to the production of large quantities of stable protein once the region of the amino acid sequence that yields high expression is determined [10]. In this approach, each protein domain is maintained intact and minor changes are made to the length of the construct at either the N and/or C-termini. Here, we present a comprehensive high-throughput study using over 200 hSTIM1 constructs to identify the key domains and boundaries that must be maintained to obtain a high yield of stable and homogeneous hSTIM1. This multi-construct approach has also revealed constructs that were not expressed properly and therefore should be avoided. Finally, to validate the integrity of high quality constructs, we have performed surface plasmon resonance (SPR), nuclear magnetic resonance (NMR) and thermostability analyses, which demonstrate the functionality of the resulting constructs.

## Results and Discussion

### Expression of hSTIM1

**Figure 1:**
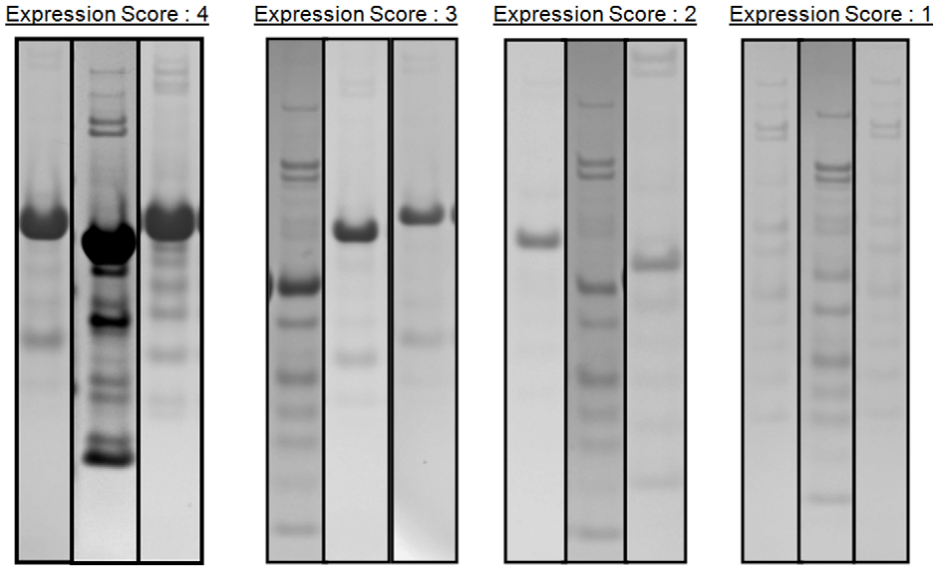
Coomassie stained gel images of selected hSTIM1 constructs. The hSTIM1 constructs are scored from a range of 1 to 4: An expression score of ‘4’ indicates high expression of target protein relative to gel background with the appropriate MW; ‘3’ indicates high expression of target protein relative to gel background with the appropriate MW; ‘2’ indicates medium to low expression of target protein relative to gel background with the appropriate MW; ‘1’ indicates either the absence of expression of target protein or an incorrect MW representation.

A total of 211 hSTIM1 constructs were designed based on the key regions of wild-type hSTIM1. Within each key region, a number of highly similar constructs were designed that differ only slightly in their lengths (1–2 amino acids difference) to identify the exact regions that could yield stable and homogeneous protein constructs for further biophysical characterization and crystallization. Table 1 summarizes the results for the screen of the expression of these constructs. A total of 196 constructs were successfully cloned and expressed of which 30 contained both the N-terminal and the transmembrane region. All the constructs that contained the N-terminal region were truncated and began from S58 because this truncation was previously shown to yield the most stable N-terminal constructs [9]. The successfully cloned constructs were further sorted by scoring them from 1–4 based on their observed expression level, with ‘4’ indicating a high expression level, ‘3’ good expression, ‘2’ low expression and ‘1’ very low or no expression. The expression levels were determined empirically through the estimation of the observed protein level by SDS-PAGE followed by Coomassie staining (Fig. 1). Using this scoring system, over 70% of the constructs yielded good or high levels of expression (Table 1). Nevertheless, some of the constructs were found to contain high levels of impurities and possible degradation products (Fig. 1).

**Table 1:** Expression scores for the hSTIM1 constructs.

Constructs	Expression Score				
	4	3	2	1	Failed
**Transmembrane**	23	6	1	0	3
**Soluble**	70	55	17	28	8
**Total**	93	61	18	28	11
**Percentage**	44%	29%	9%	13%	5%

An expression score of ‘4’ indicates high expression, ‘3’ indicates good expression, ‘2’ indicates low expression and ‘1’ indicates very low expression. Overall, majority of soluble hSTIM1 constructs expressed well with 73% having good to high expression.

### Expression analysis of the transmembrane-containing constructs

**Figure 2:**
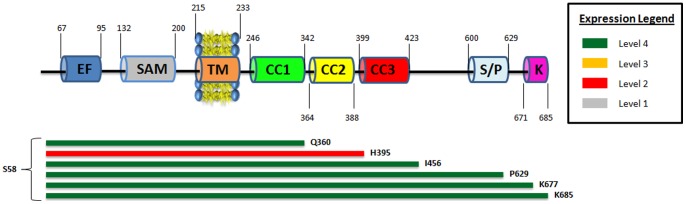
The relative expression levels of various transmembrane hSTIM1 constructs. Most of the transmembrane constructs expressed very well. The amino acid identity of both the start and end residues are displayed at the end of each color bar. Green indicates an expression score of 4; Yellow indicates an expression score of 3; Red indicates an expression score of 2. Grey indicates an expression score of 1. A graphical representation (not to scale) of wild-type hSTIM1 protein is embedded at the top for easier reference to the key domains with the approximate amino acid numbers listed: EF hand (EF), the sterile-α motif (SAM) domain, transmembrane region (TM), three coiled-coil domains (CC1, CC2 and CC3), serine/proline rich region (S/P) and the lysine rich region (K).

The expression level of the transmembrane-containing constructs was determined by SDS-PAGE analysis, following small-scale extraction and purification with the detergent Fos-Choline-12 (FC12). This detergent was previously shown to be very efficient for the extraction and subsequent identification of low-expressing membrane proteins [11]–[13]. Because this strong detergent can solubilize even unfolded membrane proteins, which might result in false positive data [14], the quality of any membrane protein purified with FC12 must be further verified with less harsh detergents. As shown previously, a detergent screen is typically required to identify the optimal extraction and purification condition for the target membrane protein [12]. Such detailed analysis of the transmembrane-containing constructs of hSTIM1 was excluded from this study. Hence, the scoring of such constructs is solely based upon the level of the small-scale FC12-extracted construct with the correct molecular weight, as judged by SDS-PAGE. The relative expression levels of the transmembrane protein constructs are shown in Figure 2. Most of the transmembrane-containing constructs were found to express very well. The main interesting observation from this expression analysis was the decreased expression of the constructs containing the S600-K685 region, which is the Ser/Pro and Lys-rich domain (S600-K685).

### Constructs beginning at K246 yield a high level of expression

**Figure 3:**
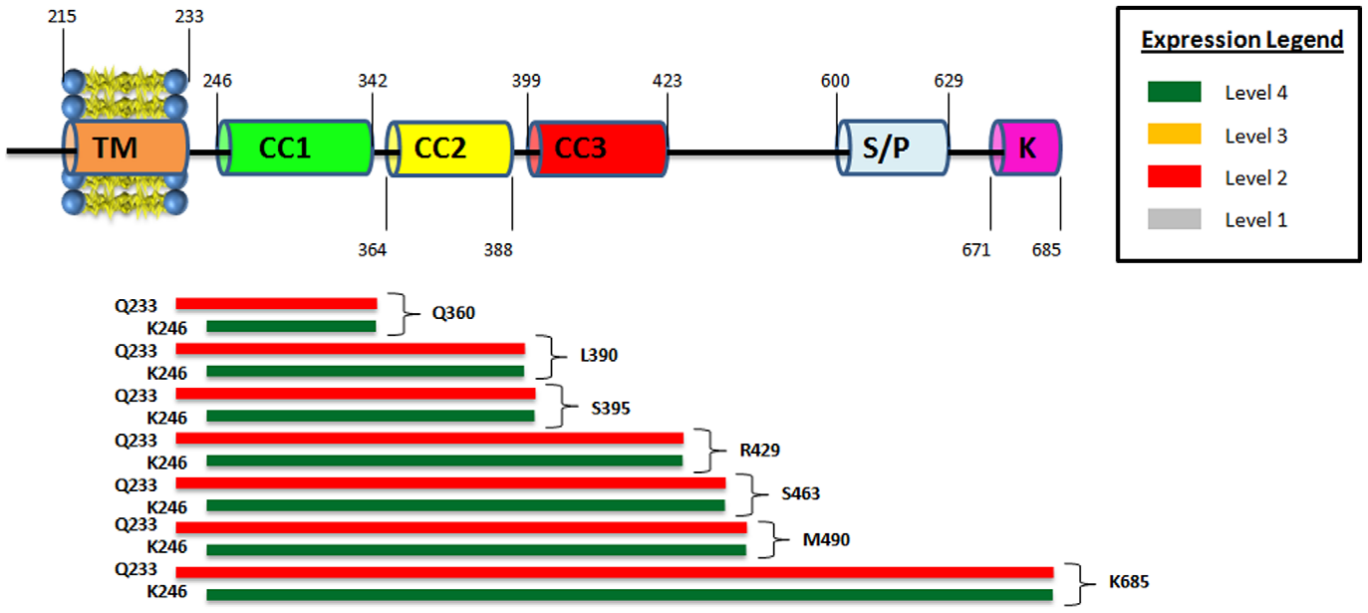
Comparison of expression scores for hSTIM1 constructs starting with either Q233 or K246. Same representation as in Fig. 2. Note that constructs starting from K246 have better expression compared to those starting from Q233 when having the same end residue.

**Figure 4:**
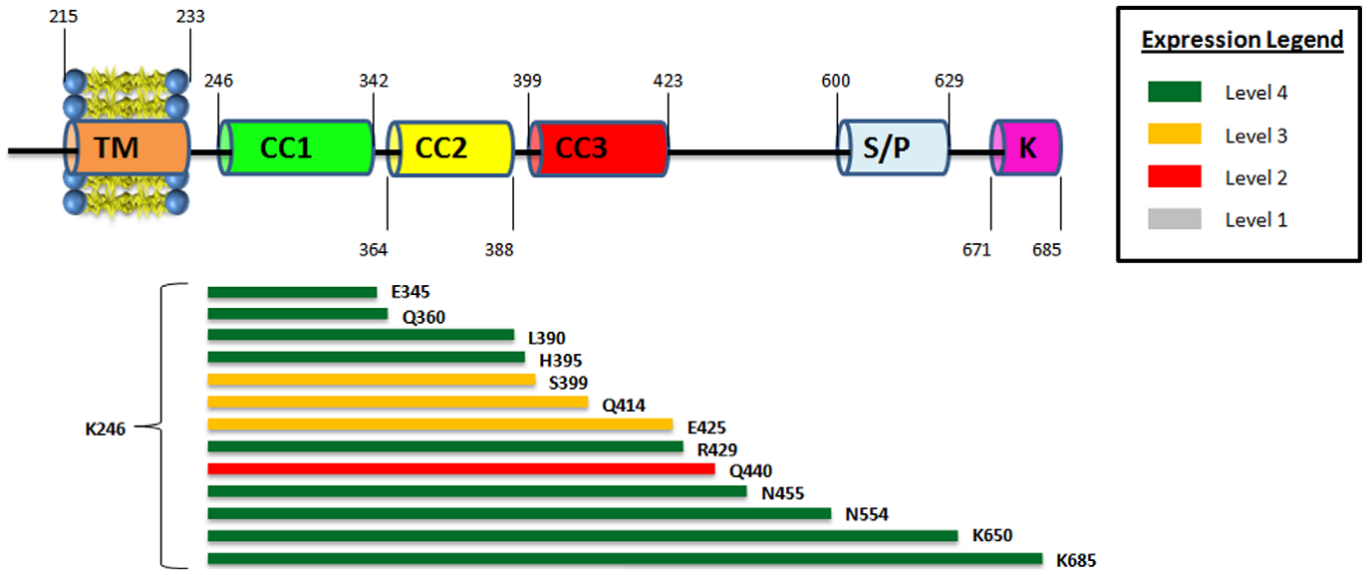
Comparison between hSTIM1 constructs that have identical starting points with different end regions. Same representation as in Figs. 2 and 3. Note that expression score dropped once there is a truncation of residues within the coiled-coil 2 and coiled-coil 3 regions.

Two similar sets of hSTIM1 C-terminal constructs, one beginning at Q233 and the other beginning at K246, were cloned, and their expression levels were analyzed. Each set consisted of seven constructs that differed in their lengths. Q233 is predicted to be the first amino acid on the cytosolic side, whereas K246 corresponds to the beginning of the first predicted coiled-coil region. The expression and quality studies of these fourteen constructs revealed that all the constructs beginning at K246 yielded a very high level of expression, in contrast to those beginning at Q233 (Fig. 3). We expanded the number of constructs beginning at K246, and the majority showed very high levels of expression (Fig. 4). Thus, constructs beginning at K246 yield high levels of C-terminal hSTIM1 fragments. Currently, it is unclear why the Q233-constructs demonstrated much less expression compared with those beginning at K246. It is possible that the Q233-K246 region makes the protein more flexible or less stable, which leads to higher levels of inclusion bodies or severe degradation of the hSTIM1 constructs. Most of the reported C-terminal constructs have been designed to begin at position Q233, but the expression was performed in eukaryotic cells that typically resulted in fairly low levels of expression [15]. Note that we were able to obtain the expression of all constructs beginning at Q233, but the level of the expression was low in comparison to the K246 constructs. Therefore, it would be interesting to investigate whether C-terminal constructs beginning at K246 would also yield much higher expression levels in eukaryotic cells.

### The first coiled-coil domain is required for stable hSTIM1 expression

**Figure 5:**
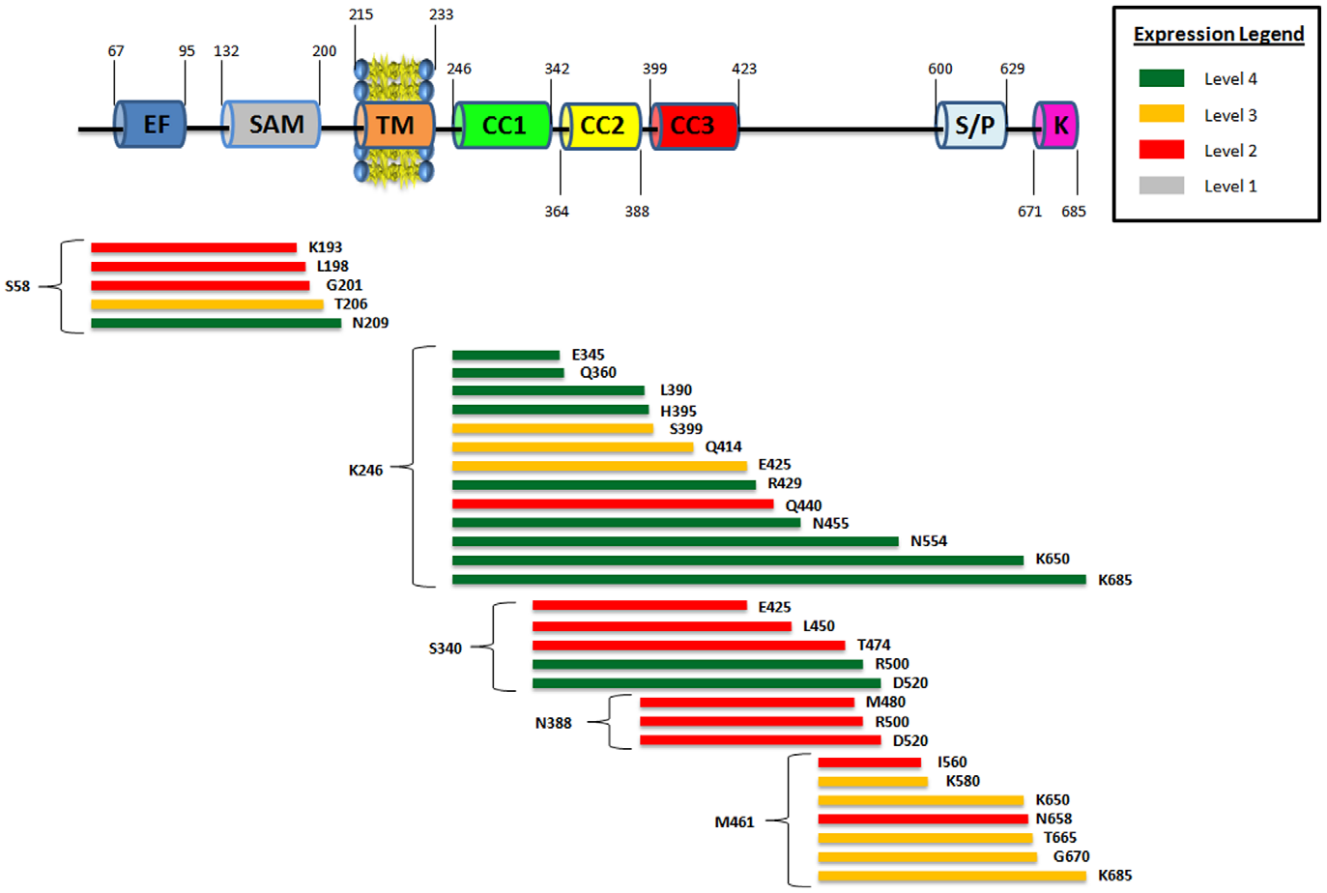
Relative expression scores of various soluble hSTIM1 constructs. Same representation as in Figs. 2, 3, 4. Note that lower expression levels were observed for these hSTIM1 constructs that do not contain any coiled-coil regions compared to constructs with the entire coiled-coil domain region.

The large cytosolic domain of hSTIM1 is distinguished by the high content of coiled-coil forming helical regions. Three coiled-coil domains have been predicted within the regions K246-Y342, I364-N388 and S399-L423. The channel activating domain (CAD) [5] and the inhibitory domain are found within this region [5], [16], [17]. Therefore, this region is of special interest when studying the mechanism of hSTIM1 function. As outlined in Figure 4, nearly all constructs containing the entire coiled-coil region demonstrated a high level of expression. However, there was a clear decrease in the expression level when the constructs were made slightly shorter than the coiled-coil region. To investigate the effect of the absence/presence of each coiled-coil domain on the expression level of hSTIM1 fragments, new constructs were designed beginning from either coiled coil 2 (S340) or coiled coil 3 (N388). The latter had a detrimental effect on the expression level of the constructs (Fig. 5). The constructs beginning at S340 yielded a high level of expression only when an extended region beyond the coiled-coil domains was included. Compared with the constructs beginning at K246, the overall yield of the two sets of coiled-coil constructs was much lower. Complete removal of the coiled-coil domains yielded proteins with good expression levels (Fig. 5). Hence, we could conclude that to obtain stable hSTIM1 C-terminal fragments, the presence of an intact coiled-coil region, especially coiled coil 1, is necessary. This conclusion was further strengthened by the increased stability of constructs containing coiled coil 1 compared with those lacking it, as shown by the results of the thermostability shift assay (Table 2).

**Table 2:** hSTIM1 melting temperature (T_m_) comparison between constructs with different start regions and identical end points.

Start residue	End residue	Coiled-coil regions	Melting Temperature (T_m_)/°C	T_m_ Difference*
**246**	516	1–3	56.7	9.7
**340**	516	2–3	47.0	
**246**	514	1–3	54.4	7.2
**340**	514	2–3	47.2	
**246**	500	1–3	53.7	7.0
**340**	500	2–3	46.7	
**246**	490	1–3	53.4	6.6
**340**	490	2–3	46.8	

The difference in their melting temperatures is tabulated at the end and is colored green. Constructs were found to have a higher T_m_ when the first coiled-coil region (246–342) was present.

* between constructs with identical end points.

### Identification of high quality hSTIM1 constructs

**Figure 6:**
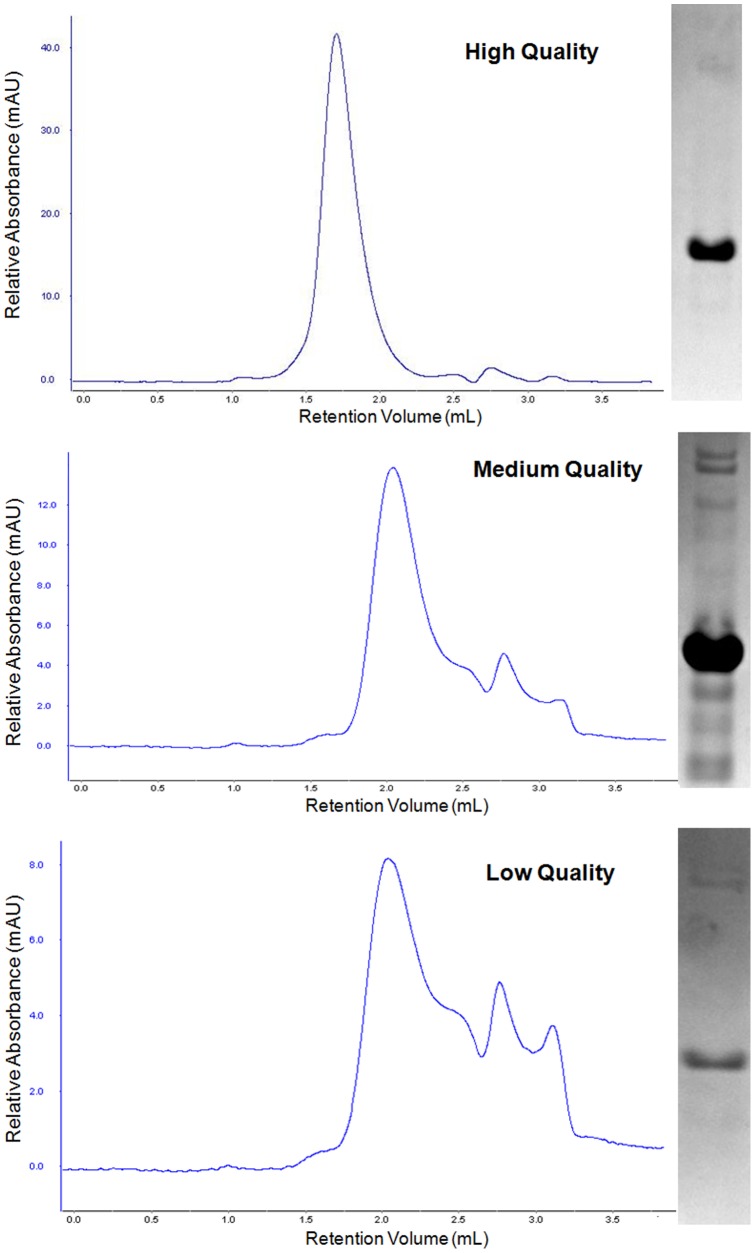
Analytical Size Exclusion chromatograms and SDS-Page gel electrophoresis photos of selected hSTIM1 constructs. The scoring of the chromatograms is differentiated into High, Medium and Low quality. The chromatograms are plotted as relative absorbance (mAU) against retention volume (mL). Chromatograms of high quality constructs have a dominant peak area of >80% of the entire peak area. Chromatograms of medium quality constructs quality have a dominant peak area of >60% but <80% of the entire peak area. Chromatograms of low quality constructs have multiple peaks present. Their corresponding SDS-Page gel photos are placed next to the chromatograms.

Next, we identified hSTIM1 constructs that are of high quality and useful for biochemical and biophysical studies. To achieve this goal, we used analytical size exclusion chromatography (ASEC) to ascertain the promising constructs. ASEC has proven to be a highly reliable technique to verify the quality of protein preparations, which is typically scalable [18], [19]. Thus, samples that were originally scored as 3 or 4 were further analyzed by ASEC. Here, the chromatograms were the main factors in determining the protein quality. As shown in Figure 6, high quality samples are referred to as highly homogenous samples judged by both SDS-PAGE and ASEC, whereas low/medium quality samples contain impurities and are heterogeneous. After analyzing the constructs, the high quality constructs were identified and are summarized in Table 3.

**Table 3:** List of hSTIM1 constructs that have both the highest expression score and quality out of the 211 constructs that have been cloned and expressed.

*Number*	*Start residue*	*End residue*
**1**	58	520
**2**	58	557
**3**	58	580
**4**	58	600
**5**	246	345
**6**	246	360
**7**	246	490
**8**	246	500
**9**	246	520
**10**	246	556
**11**	246	561
**12**	246	567
**13**	246	571
**14**	246	575
**15**	246	580
**16**	246	587
**17**	246	595
**18**	246	600
**19**	246	632
**20**	246	650
**21**	246	660
**22**	246	667
**23**	246	685
**24**	340	490
**25**	340	520
**26**	340	556
**27**	340	571
**28**	340	575
**29**	340	580
**30**	340	685

Expression score was determined based on protein expression in SDS-Page gel electrophoresis analysis. Quality score was determined based on Analytical Size exclusion chromatogram profile.

### SEC at low ionic strength improves the quality of purified hSTIM1 constructs

**Figure 7:**
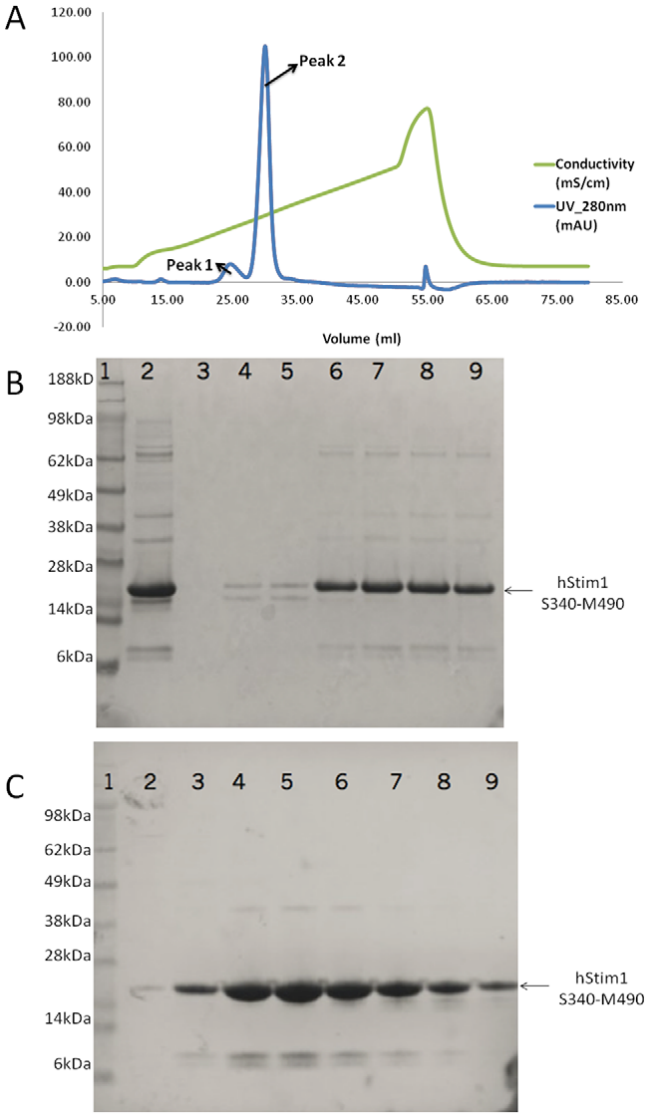
Ion exchange (**IEX**) **chromatogram and SDS-PAGE photos of hSTIM1 S340-M490 purification.** **A**: The UV signal at 280 nm (UV_280 nm) is plotted relative absorbance (mAU) against retention volume (mL) as shown in blue. The corresponding salt concentration is plotted as conductivity (mS/cm) against retention volume (mL) as shown in green. On UV_280 nm plot, two peaks were separated. Fractions from Peak1 and Peak2 were analyzed by SDS-PAGE as shown in **B**: The most prominent band represents protein of interest. 1, protein marker; 2, injected sample before IEX; 3, empty; 4 and 5, fractions from peak 1; 6–9, fractions from peak 2. Fractions from peak 2 were pooled and subsequently purified by size exclusion chromatography (SEC) with low salt (50 mM NaCl) buffer. SDS-PAGE analysis of the protein content after SEC is shown in **C**: 1, protein marker; 2–9, fractions of elution peak from SEC.

For constructs lacking coiled coil 1, we noticed that the increased instability and decreased solubility of the protein correlated with increased impurities. Hence, we included ion exchange chromatography (IEX) as a subsequent step to immobilized metal affinity chromatography (IMAC) and size exclusion chromatography (SEC). When purifying the CAD-containing construct (S340-M490), two peaks were separated by IEX, with the peak that eluted with a higher salt concentration (Peak 2) as the most prominent (Fig. 7A). SDS-PAGE analysis of the protein content of Peak 2 revealed that the hSTIM1 construct was eluted together with a prominent portion of impurities (Fig. 7B). The target protein appeared to elute with a conductivity of approximately 30 mS/cm (equivalent to approximately 300 mM NaCl), i.e., at relatively high ionic strength. Taking into consideration the coiled-coil rich nature of this construct, it is possible that the protein would precipitate in a high ionic condition as a result of promoted protein-protein hydrophobic interactions. In this particular case, the impurities may associate with the target protein via hydrophobic interactions. To test this hypothesis, the eluate from Peak 2 was subjected to SEC at a low ionic strength (50 mM NaCl). SDS-PAGE analysis of the eluates from this low salt SEC step (Fig. 7C) reveals a clear improvement in the purity of the hSTIM1 construct compared with those that were obtained from IEX. Additionally, the solubility of this construct increased more than twenty fold from 70 μM to 1.6 mM, which indicates increased stability. The introduction of a similar low salt SEC step into the purification of other hSTIM1 constructs lacking coiled coil 1 resulted in increased purity and solubility as well (data not shown).

The coiled coil 1 domain has been reported to be involved in the autoinhibition mechanism of hSTIM1 activation [16], [17]. Through this autoinhibition mechanism, a weak electrostatic interaction between the CAD and the coiled coil 1 domain of hSTIM1 appears to mask the CAD when hSTIM1 is in a resting state. Hence, activation of hSTIM1 would require unmasking the CAD region, which could be achieved by a conformational change in hSTIM1 in response to Ca^2+^ loss. Therefore, constructs lacking coiled coil 1 would contain an exposed CAD, possibly mimicking the active state of hSTIM1. With the CAD domain exposed, the probability of binding other contaminant proteins could also increase. In contrast, constructs containing the coiled coil 1 domain would mimic hSTIM1 in its resting state, in which coiled coil 1 masks the CAD domain. Hence, the increased stability and purity of constructs containing coiled coil 1 can be attributed to the masking of CAD by coiled coil 1 that would prevent the interaction between hSTIM1 and impurities.

### Monitoring the hSTIM1-Orai1 interaction with surface plasmon resonance (SPR)

**Figure 8:**
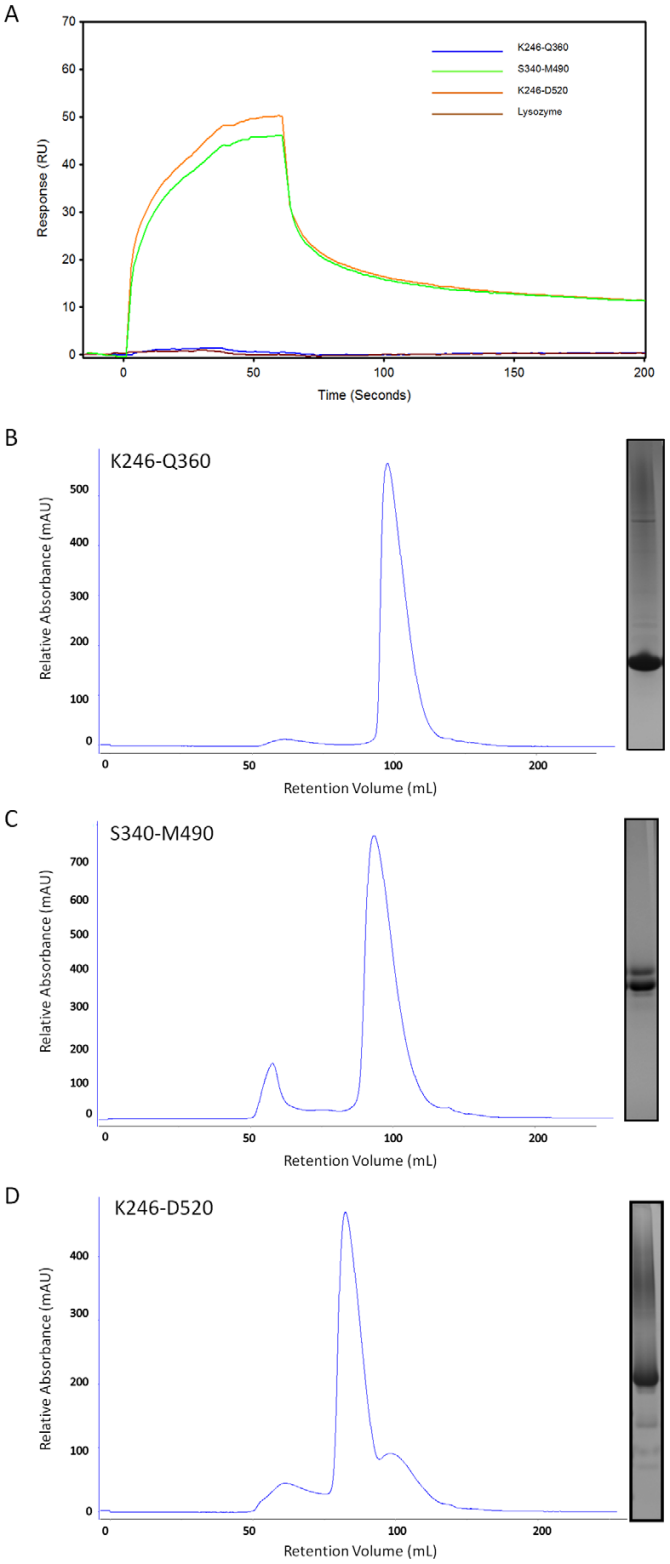
SPR sensorgrams with their corresponding ASEC and gel images of selected hSTIM1 constructs. **A**: All hSTIM1 constructs injected have a concentration of 10 µM. The Orai1 peptide was immobilized at 1000 RU for all runs. Lysozyme is included as negative control. SPR sensorgrams are plotted as response units (RU) against time (seconds). **B–D**: Analytical Size Exclusion chromatograms and SDS-Page gel electrophoresis of K246-Q360 (**B**); S340-M490 (**C**); K246-D520 (**D**). Chromatograms are plotted as relative absorbance (mAU) against retention volume (mL).

To verify the functionality of the high quality samples, we performed SPR studies to monitor the interactions between the hSTIM1 constructs with the Orai1 C-terminus. The Orai1 C-terminus was synthesized with the addition of an extra Cys at the N-terminus to immobilize it on a carboxymethylated dextran matrix via thiol coupling. Such coupling leaves a free C-terminal end that mimics the *in vivo* topology of the Orai1 C-terminus. We monitored the interactions between the immobilized Orai1 C-terminus using three different hSTIM1 constructs: coiled coil 1 only (K246-Q360), the CAD (S340-M490) and K240-D520 in which the entire coiled-coil domain, including the CAD, were present. These constructs were chosen as their overall electronegativity and hydrophobicity are rather similar. Therefore, under identical experimental conditions (i.e., a constant level of immobilized Orai1 C-terminus in all experiments, an identical concentration of hSTIM1 constructs and an identical buffer condition) any significant differences in the interactions with the Orai1 C-terminus could reflect specific interactions. At this point, SPR was only used to probe the proximity interaction between the Orai1 C-terminus and hSTIM1 constructs. As shown in Figure 8, no interactions between the coiled coil 1 (lacking the CAD) and the Orai1 C-terminus were observed, whereas the CAD-containing hSTIM1 constructs and the Orai1 C-terminus demonstrated strong responses. Lysozyme was used as a negative control and, similar to the coiled coil 1 (K246-Q360) construct, demonstrated no response when injected over the identical immobilized Orai1 surface. Hence, the observed interactions between the CAD-containing hSTIM1 constructs and the Orai1 C-terminus are specific protein-protein interactions. These results strongly suggest that the hSTIM1 constructs are indeed functional, as the Orai1 C-terminus has been shown to interact only with CAD-containing constructs [20], [21].

### Characterization of hSTIM1 with nuclear magnetic resonance (NMR)

**Figure 9:**
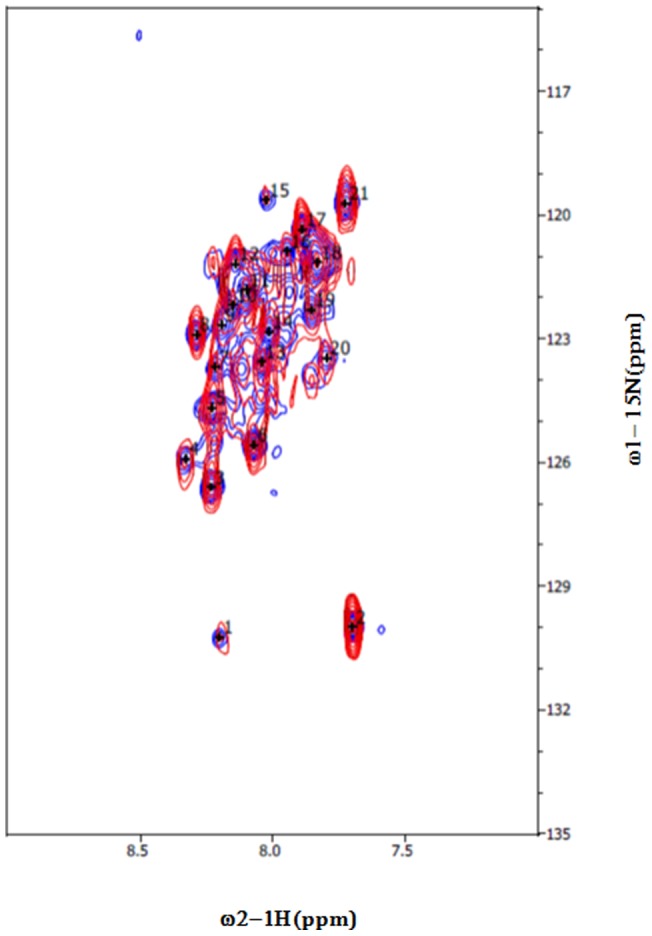
Superimposed [^15^N, ^1^H]-TROSY spectra of free hSTIM1 and hSTIM1 bound to Orai1 C-terminal derived peptide. The free hSTIM1 is colored blue and the Orai C-terminal peptide is red. The spectra were collected at 298 K in 20 mM TRIS, 300 mM NaCl, 0.1 mM TCEP at pH 8.0.

**Figure 10:**
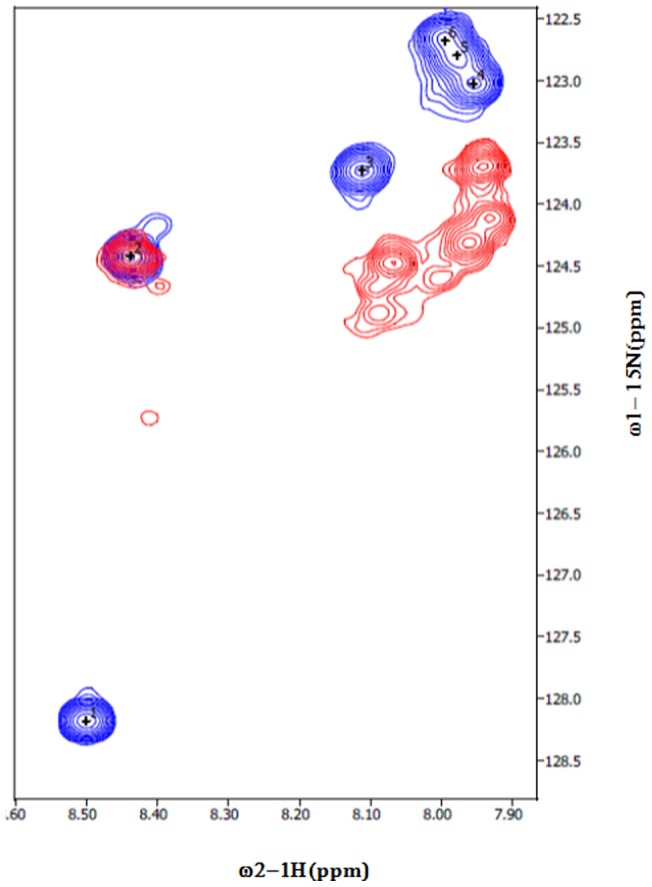
Superimposed [^15^N, ^1^H]-HSQC spectra of Orai1 derived C terminal peptide. [^15^N, ^1^H]-HSQC of free peptide (colored blue) contains specifically ^15^N labeled Leu. [^15^N, ^1^H]-HSQC of peptide with unlabeled hSTIM1 K246-S600 is colored red. The spectra were collected at 298 K in 20 mM HEPES, 300 mM NaCl, 0.1 mM TCEP at pH 8.0.

To characterize the binding of hSTIM1 coiled-coil domains with the peptides derived from the Orai1 C-terminal region, we recorded the [^15^N, ^1^H]-TROSY spectrum of hSTIM1 (K246–S600) and monitored the chemical shift perturbations of hSTIM1 resonances upon titration with the Orai1-derived peptide. Complete residue specific assignment of the F resonances was not achieved due to significant line broadening on a majority of the resonances. This finding is presumably a result of pervasive conformational exchange. However, resonances in TROSY spectra were still observed and tentatively assigned to the terminal regions of hSTIM1. Upon titration with an Orai1-derived peptide, changes in the position of cross-peaks of hSTIM1 were observed (Fig. 9). The majority of the resonances stemming from the Orai1 peptide were significantly perturbed compared to its free state, indicating the acquisition of secondary structure in the otherwise unstructured peptide. Figure 10 illustrates the spectral perturbations caused by the addition of the peptide, indicating interactions between the CAD-containing hSTIM1 and the Orai1 peptide.

In conclusion, the presented report based on the comprehensive analyses of hSTIM1 regions indicates three key factors for obtaining large quantities of stable hSTIM1 constructs: 1) K246 is the most suitable initial amino acid for soluble cytosolic constructs, 2) the first coiled-coil region is required to obtain stable constructs, and 3) SEC using a buffer of low ionic strength improves the quality of the obtained constructs. In addition, the wealth of various constructs produced and characterized in this study identifies the suitable constructs, which we have shown to be functional by various biophysical assays, for in-depth structural and functional investigations of this protein.

## Materials and Methods

### Cloning of hSTIM1 constructs

The hSTIM1 constructs were cloned into the expression vector pNIC-28-*Bsa*I by ligation independent cloning (LIC) to introduce an additional DNA encoding for an N-terminal hexa-histidine tag paired together with a TEV protease cleavage site (MHHHHHHSSGVDLGTENLYFQSM), as previously described [22]. The genes were amplified by PCR with an equal mixture of both Phusion™ High-Fidelity DNA Polymerase (Finnzymes) and Platinum® Pfx DNA Polymerase (Invitrogen) following the manufacturer's recommendation.

The PCR products were subsequently purified using the PureLink Pro 96 PCR Purification Kit (Invitrogen) and treated with T4 DNA polymerase in the presence of dCTP to generate sticky ends. The pNIC-28-*Bsa*I vector was linearized using the restriction enzyme *Bsa*I and further treated with T4 DNA polymerase in the presence of dGTP to generate sticky ends complementary to the PCR products. This step was followed by an annealing step and transformation into *E. coli* MACH1 cells. Colonies were subsequently grown in Luria-Bertani broth (LB) agar plates containing 50 µg/mL kanamycin and screened for correctly sized inserts by PCR using the BIOTAQ™ Red DNA Polymerase (Bioline). Plasmids from the positive colonies were transformed into *E. coli* Rosetta cells (DE3). Colonies were then grown on LB agar plates containing 50 µg/mL kanamycin and 34 µg/mL chloramphenicol.

### Small-scale expression screen

Overnight cultures were diluted 1∶100 in 1 mL of Terrific Broth (TB) medium (Formedium, United Kingdom) and grown at 37°C in 96-well deep-well plates by shaking at 220 rpm until the OD_600_ reached 0.8. Protein expression was induced with 0.2 mM of isopropy1-β-D-thiogalactopyranoside (IPTG), and the cultures were further grown overnight at 18°C. The cells were then harvested by centrifugation at 3200× *g* for 5 minutes. The cells pellets were resuspended in lysis buffer (20 mM HEPES, pH 8.0, 150 mM NaCl, 30 mM imidazole) supplemented with a cocktail of protease inhibitors (Roche, Switzerland), 1 mg/mL lysozyme (Sigma-Aldrich, USA) and 10 units/mL Benzonase (Merck, USA). The cells were then lysed by three cycles of freezing at −80°C and thawing at 25°C. The lysate was clarified by centrifugation at 3200× *g* for 15 minutes through a 96-well filter plate (0.65 µm) (Millipore, USA). The filtrate was then transferred to a 96-well filter plate containing Ni-NTA agarose resin (Invitrogen) for purification using IMAC. After 15 min of incubation at 4°C, the unbound material was removed by centrifugation at 100× *g* for 30 s. The wells were then washed with 10 column volumes (CV) of wash buffer (20 mM HEPES, pH 8.0, 150 mM NaCl, 30 mM imidazole) by centrifugation at 100× *g* for 1 min. The bound recombinant proteins were then recovered in 30 μL of elution buffer (20 mM HEPES, pH 8.0, 150 mM NaC1, 300 mM imidazole) by centrifugation at 100× *g* for 1 min. The eluted material was analyzed by SDS-PAGE using NuPAGE 4%–12% Bis-Tris gels (Invitrogen) and stained with SimplyBlue Coomassie stain (Invitrogen). Purified protein constructs were subsequently loaded onto a Superdex 200 5/150 analytical size exclusion column (GE Healthcare) connected to an AKTAmicro chromatography system controlled by Unicorn software version 5.20 (GE Healthcare).

### Large-scale protein purification

The overnight cultures were diluted 1∶500 in 1 L of TB (Formedium, United Kingdom). The cells were then grown further at 37°C by shaking at 220 rpm until the OD_600_ reached 0.6–0.7. Protein expression was induced by the addition of 0.2 mM IPTG and grown overnight at 18°C. The cell cultures were harvested by centrifugation at 10,000× *g* for 10 min, and the pellets were resuspended in lysis buffer supplemented with a cocktail of protease inhibitors (Roche, Switzerland), 1 mg/mL lysozyme (Sigma-Aldrich, USA) and 10 units/mL Benzonase (Merck, USA). The cells were lysed by three cycles of freezing at −80°C and thawing at 25°C. Unbroken cells and cell debris were removed by centrifugation at 30,000× *g* for 20 minutes. The supernatant was loaded onto 1 mL of Ni-NTA agarose beads (Invitrogen, USA), washed with 10 CV of wash buffer and then eluted with elution buffer. Purified protein constructs were then injected onto either a Superdex 75 or Superdex 200 16/60 size exclusion column (GE Healthcare). Prior to IEX, the samples were desalted using a PD-10 desalting column (GE Healthcare) into an initial buffer (20 mM Tris-HCl, pH 8.0, 50 mM NaCl, 5 mM TCEP). The desalted samples were subsequently injected into a pre-equilibrated 1 mL HiTrap Q HP column (GE Healthcare, USA). A linear NaCl gradient from 50 mM to 1 M NaCl in an identical buffer was used for the elution of the target protein. All IEX and SEC were performed using an AKTAexplorer FPLC chromatography system controlled by Unicorn software version 5.20 (GE Healthcare), and the fractions were analyzed by SDS-PAGE using NuPAGE 4%–12% Bis-Tris gels (Invitrogen) and stained with SimplyBlue Coomassie stain (Invitrogen).

### Production and sequence of the Orai1 C-terminal domain

The Orai1 C-terminal domain was synthesized by Peptide 2.0 (USA) and delivered in a lyophilized state. The sequence of the Orai1 C-terminal peptide is as follows: CRSLVSHKTDRQFQELNELAEFARLQDQLDHRGDHPLTPGSHYA.

A Cys was added to the N-terminus of the Orai1 peptide to enable immobilization via thiol coupling to a CM5 chip (GE Healthcare). The lyophilized peptides were dissolved in acidic buffer (10 mM sodium acetate, pH 6.0) and injected onto a Superdex Peptide 10/300 size exclusion column (GE Healthcare) connected to an AKTAmicro controlled by Unicorn software version 5.20 (GE Healthcare). The quality of the fractions was analyzed by SDS-PAGE using NuPAGE 4%–12% Bis-Tris gels (Invitrogen) and stained with SimplyBlue Coomassie stain (Invitrogen). The purified peptides (approximately 100 µM) were then stored in 100 µL aliquots at −20°C.

### SPR analysis of hSTIM1 constructs

The binding of different hSTIM1 constructs to Orai1 peptides (synthesized by Peptide 2.0) was assessed on a Biacore T200 (GE Healthcare). Orai1 peptide was immobilized on a CM5 sensor chip (GE Healthcare) by ligand thiol coupling. The surface was activated using 20 µL of a 1∶1 mixture of 0.4 M 1-ethyl-3-(3-dimethylaminopropyl)-carbodiimide (EDC) and 0.1 M N-hydroxysuccinimide (NHS) with a running flow rate of 10 µL/min. Reactive disulfide groups were introduced to the surface with an injection of 80 mM 2-(2-pyridinyldithio)ethaneamine (PDEA) in 0.1 M sodium borate, pH 8.5, at a running flow rate of 10 μL/min for 4 min. The Orai1 peptide (approximately 100 µM) was injected at a flow rate of 10 μL/min until an immobilization of 1,000 RU was obtained. Twenty microliters of 50 mM cysteine and 1 M NaCl in 0.1 M sodium acetate buffer, pH 4.3, at a running flow rate of 5 μL/min was subsequently injected for 4 min to deactivate any remaining reactive groups. Blank immobilization was performed on the first flow cell, which was used as a reference flow cell for subsequent analysis. Various hSTIM1 constructs at concentrations of 10 µM were injected over the sensor surfaces with HBS-EP buffer (20 mM HEPES, pH 7.4, 150 mM NaCl, 3.4 mM EDTA, 0.005% Polysorbate 20) at a flow rate of 30 μL/min for 1 min, allowing 4 min for the dissociation before regeneration. The interactions between the ligand and the analyte were measured over a 6-min time period allowing for a dissociation time of four minutes. Between injection cycles, 10 mM glycine-HCl pH 3.5 was used to regenerate the surface with a contact time of 30 sec. Lysozyme (approximately 10 µM) was injected as a negative control. SPR data were analyzed using Scrubber software (v2.0c, Biologic, Australia).

### Protein Expression and Purification for NMR studies

Constructs of hSTIM1 containing the domains coiled coil 1, 2 and 3 with an extension (K246-S600) were used for this study. ^13^C, ^15^N-isotope labeled hSTIM1 protein was obtained by growing *E. coli* cells in M9 minimal media containing isotope labeled glucose and NH_4_Cl (Cambridge Isotope Laboratories). Induction was performed using 0.5 mM IPTG for approximately 18 hours at 18°C and purified using identical chromatographic procedures as described previously. The Orai1 C-terminus derived peptide (China Peptides) contained an identical sequence used previously except for the presence of six ^15^N-labeled Leu.

### NMR Sample Preparation

The sample used for the NMR study consisted of 0.3 mM ^15^N-labeled hSTIM1 in 90% H_2_O/10% D_2_O. All samples used to record the two-dimensional TROSY were prepared in 20 mM Tris-HCl and 300 mM NaCl adjusted to pH 8.0. Identical buffer conditions were used for the binding studies of hSTIM1 with the Orai1 C-terminus-derived peptide. The peptide was prepared as a 50 mM stock solution in an identical buffer as that for the hSTIM1 protein. The addition of the Orai1 C-terminus-derived peptide to the peptide-free hSTIM1 sample was performed stepwise to achieve a final concentration of Orai1 peptide: hSTIM1 protein with a ratio of 1∶1. The hSTIM1 sample without any Orai1 peptide was used as the reference for observing any chemical shifts. All NMR experiments were conducted at 298 K.

### NMR Spectroscopy

All NMR experiments were performed on a Bruker AVANCE II 600 MHz and 700 MHz NMR Spectrometer equipped with four RF channels and a 5 mm Z-gradient TCI CryoProbe. All NMR experiments were performed at 298 K, and sweep widths for ^1^H and ^15^N were 9804 and 2412 Hz, respectively. Two-dimensional [^15^N,^1^H]-TROSY was performed for each sample and binding experiments. Data were processed using Topspin 2.1, and the chemical shifts were referenced directly (^1^H) to the frequency of DSS. Peak picking and spectral analysis was performed using CARA.

### Thermal shift assay

The thermostability of the hSTIM1 constructs was studied using differential static light scattering as described by Hong et al. [23] using a multiwell instrument (Stargazer, Harbinger Biotech, Toronto, Canada). Protein samples with concentrations of 30 µM in 50 μL of buffer (20 mM HEPES, pH 7.5, 300 mM NaCl) were heated from 25–80°C at a rate of 1°C/min. The aggregation of protein was determined through the recording of scattered light using a CCD camera and images of the plate were taken at every 0.5°C interval. Using proprietary software, pixel intensities of selected wells were integrated, thereby obtaining an approximate value that is representative of the total amount of scattered light in that particular region. The total intensities were then plotted against temperature and fitted to the Boltzmann equation by nonlinear regression. The temperature it takes for 50% of the studied protein to achieve the aggregation state was defined as the melting temperature, T_m_.
